# Chemistry of Organophosphonate Scale Growth lnhibitors: 2. Structural Aspects of 2-Phosphonobutane-1,2,4-Tricarboxylic Acid Monohydrate (PBTC.H_2_O)

**DOI:** 10.1155/BCA.2005.119

**Published:** 2005

**Authors:** Konstantinos D. Demadis, Raphael G. Raptis, Peter Baran

**Affiliations:** 1 Department of Chemistry University of Crete 300 Leoforos Knossos Crete Heraklion GR-71409 Greece; 2 Department of Chemistry University of Puerto Rico-Río Piedras P.O.Box 23346 San Juan PR 00931-3346 Puerto Rico

**Keywords:** phosphonates, carboxylates, PBTC, hydrogen bonding, scale inhibitor

## Abstract

Industrial water systems often suffer from undesirable inorganic deposits, such as calcium carbonate, calcium phosphate(s), magnesium silicate, and others. Synthetic water additives such as phosphonates and phosphonocarboxylates are the most important and widely utilized scale inhibitors in a plethora of industrial applications. The design of efficient and cost-effective inhibitors, as well as the study of their structure and function at the molecular level are important areas of research. This study reports the crystal and molecular structure of PBTC (PBTC = 2-phosphonobutane-1,2,4-tricarboxylic acid), one of the most widely used scale inhibitors in the cooling water treatment industry. Triclinic PBTC monohydrate crystallizes in the P 1 space group with cell dimesions, a =7.671(1) Å, b = 8.680(1) Å, c = 9.886(1) Å, α = 65.518(2) deg, β = 71.683(2) deg, γ = 76.173(2) deg, V = 564.20(11) Å^3^, and Z = 2. Bond distances in the -PO_3_ moiety are 1.4928(10) Å for the P=O double bond and 1.5294(10) Å and 1.5578(10) Å for the two -P-O(H) groups. P-C and C-O bond lengths fall in the normal range. A network of hydrogen bonds are formed between the water molecule of crystallization, the -P-OH and the -COOH groups.


### Chemistry of Organophosphonate Scale Growth Inhibitors: 2. Structural Aspects of 2-Phosphonobutane-1,2,4-Tricarboxylic Acid Monohydrate ($PBTC \cdot H_2O$) §

Konstantinos D. Demadis,* Raphael G. Raptis¶, and Peter Baran¶

*Department of Chemistry, University of Crete,*
*300 Leoforos Knossos, Heraklion, Crete, Greece GR-71409*
and
*Department of Chemistry, University of Puerto*
*Rico-Rio Piedras, P.O.Box 23346, San Juan, PR 00931-3346*

### GRAPHICAL ABSTRACT

The crystal structure of PBTC, one of the most widely used scale inhibitors in cooling water treatment, exhibits a complicated network of hydrogen bonds that are formed between the water molecule of crystallization, the –P–OH and the –COOH groups.

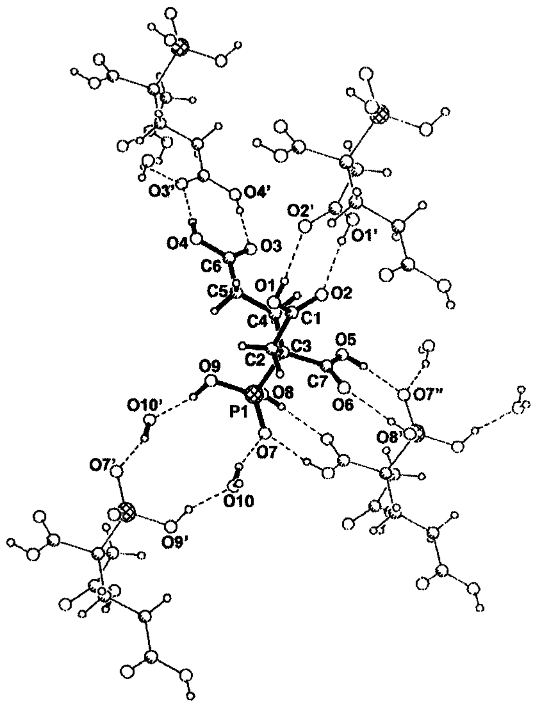

§ Part 1. Chemistry of Organophosphonase Scale Inhibitors: Two Dimensional, Layered Polymeric Networks in the Structure of Tetrasodium 2-hydroxyethy-amino-bis(methylenephosphonase). Demadis, K.D., Baran, P. J. Solid State Chem., 2004, 177, 4768.

* Author to whom correspondence should be addressed at the University of Crete. Phone: +30 2810 393651, fax: +30 2810 393601, e-mail: demadis@chemistry.uoc.gr

¶ University of Puerto Rico – Rio Piedras

### ABSTRACT

Industrial water systems often suffer from undesirable inorganic deposits, such as calcium carbonate, calcium phosphate(s), magnesium silicate, and others. Synthetic water additives such as phosphonates and phosphonocarboxylates are the most important and widely utilized scale inhibitors in a plethora of industrial applications. The design of efficient and cost-effective inhibitors, as well as the study of their structure and function at the molecular level are important areas of research. This study reports the crystal and molecular structure of PBTC (PBTC = 2-phosphonobutane-1,2,4-tricarboxylic acid), one of the most widely used scale inhibitors in the cooling water treatment industry. Triclinic PBTC monohydrate crystallizes in the $P\bar{1}$ space group with cell dimesions, a = 7.671(1) Å, b = 8.680(1) Å, c = 9.886(1) Å, $\alpha$ = 65.518(2) deg, $\beta$ = 71.683(2) deg, $\gamma$ = 76.173(2) deg, V = 564.20(11) Å$^3$, and Z = 2. Bond distances in the $-PO_3$ moiety are 1.4928(10) Å for the P=O double bond and 1.5294(10) Å and 1.5578(10) Å for the two –P–O(H) groups. P–C and C–O bond lengths fall in the normal range. A network of hydrogen bonds are formed between the water molecule of crystallization, the –P–OH and the –COOH groups.

**Keywords**: phosphonates, carboxylates, PBTC, hydrogen bonding, scale inhibitor

### INTRODUCTION

Most unsoftened (raw) industrial waters contain alkaline earth metal cations such as calcium, magnesium, barium, etc. These, particularly calcium, cause enormous problems when combined with polyanions, such as carbonate, phosphate or sulfate, and the solubility products of the corresponding salts are exceeded. The major resulting problems are precipitation and deposition of these insoluble mineral salts, unfortunately, onto the most critical equipment surfaces. Calcium carbonate /1/ and calcium phosphates /2/ are the most frequently encountered deposits. The accumulation of these deposits greatly diminishes effective heat transfer, interferes with fluid flow, facilitates corrosion processes, and can worsen microbiological fouling /3/. These phenomena are most critical in cooling water applications, where incoming water passes through a heat exchanger, cools a "hot" process and is sent back to repeat the same cooling process after it is cooled by forced evaporation /4/. This water loss by evaporative cooling results in high supersaturation levels of the above ions. Eventually, massive precipitation of sparingly soluble mineral salts can occur, either in bulk or on a surface that, in some cases, causes catastrophic operational failures.

Scale prevention can be achieved by use of scale inhibitors, key components of any chemical water treatment /6/. These are compounds (small molecules) that are added to any given treatment in minute (ppm) quantities and usually work synergistically with dispersant polymers /7/.

Phosphonates, or organic phosphates belong to a fundamental class of such compounds /8/. These usually (although not always) contain multiple phosphonate groups ($R-PO_3H_2$, R = organic chain) and are most commonly found in their deprotonated form, due to the relatively high water pH. Phosphonates are used extensively in cooling water treatment programs /9/, oilfield applications /10/ and corrosion control /11/. The structures of some common commercially available phosphonates are given in Figure 1. PBTC, HEDP (hydroxy-ethylidenediphosphonate) and AMP (amino-*tris*-methylenephosphonate) are "popular" and effective commercial scale inhibitors /12/.

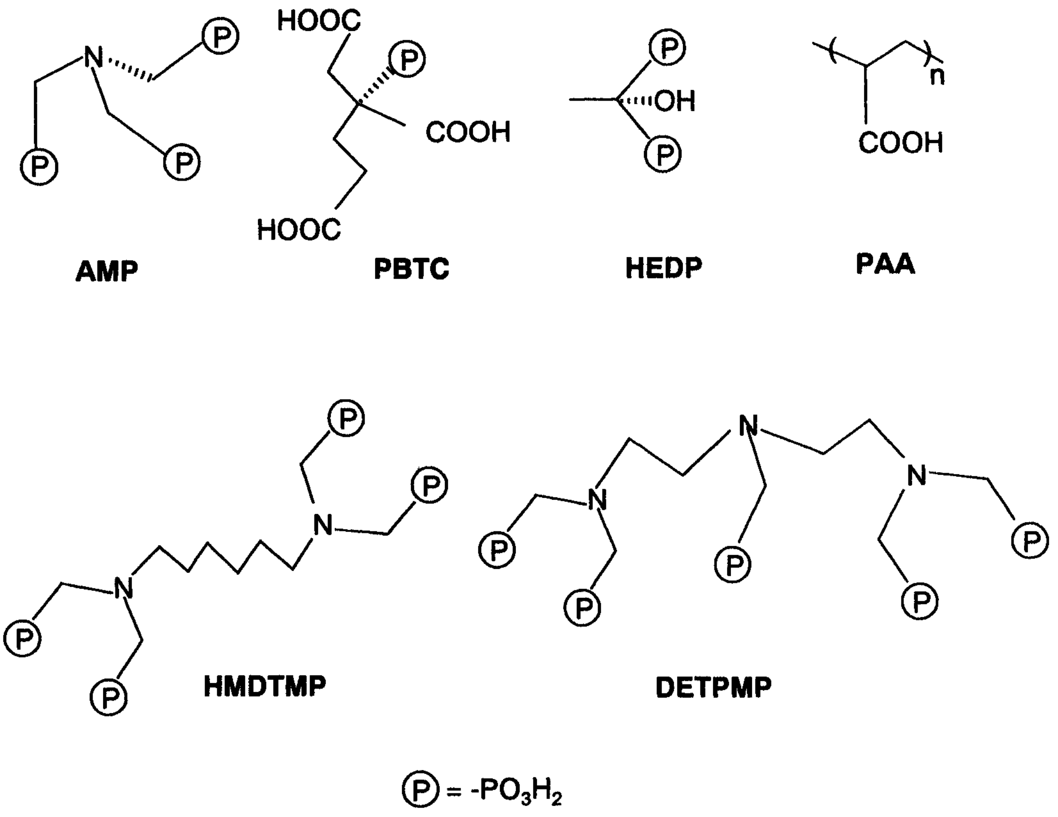

**Fig. 1:** Representative schematic structures of some representative mineral scale inhibitors. The symbol abbreviations are as follows: AMP amino-*tris*-methylene phosphonate, HEDP 1-hydroxyethylidene-1,1-diphosphonic acid, PBTC 2-phosphonobutane-1,2,4-tricarboxylic acid, PAA polyacrylic acid, HMDTMP hexamethylene-N,N',N',N'-diamine tetramethylenephosphonic acid, DETMP diethylene triaminepenta (methylenephosphonic acid).

Phosphonates are thought to achieve scale inhibition by adsorbing onto specific crystallographic planes of a growing crystal nucleus after a nucleation event. This adsorption prevents further crystal growth and agglomeration into larger aggregates /13/.

Phosphonic acids have also attracted significant attention due to their utility in supramolecular chemistry and crystal engineering /14/. Metal phosphonates commonly form pillared-layered inorganic-organic hybrid materials and microporous solids /15/. Their properties can be useful for intercalation, catalysis, sorption, and ion exchange. In these architectures hydrogen bonds are predominant resulting in one-, two, and three-dimensional supramolecular networks.

Understanding the intimate mechanisms of scale inhibition by phosphonate inhibitors requires a closer look at the molecular level of their possible function. On the other hand, structural elucidation of phosphonate "building blocks" as well as their potential supramolecular architectures may lead to the designed synthesis of new solids with novel properties. The present study aims towards this direction. In this report, the crystal and molecular structure of PBTC, 2-phosphonobutane-1,2,4-tricarboxylic acid is reported, where the presence of an extensive network of intermolecular hydrogen bonds is predominant.

### EXPERIMENTAL SECTION

### Preparations

PBTC is available in acid form under the commercial name Dequest 7000 as 50 % w/w solution in water and was used as received (Solutia UK, Newport, United Kingdom). Single crystals of PBTC·$H_2O$ suitable for crystallographic determination were obtained by slow evaporation of a concentrated solution of the compound. Crystals of PBTC·$H_2O$ formed star-like aggregates that needed to be cleaved in order to select a single crystal for structure determination. Several of those regularly shaped (rectangular plates), colorless crystals were selected, sealed in an air-tight vial (to avoid possible dehydration) for single crystal X-ray data collection.

### X-ray Structure Determination: Data Collection, Solution and Refinement of the Structure.

Relevant information concerning crystal data, intensity collection information, and structure refinement parameters for the structure are provided in Table 1. Suitable crystal was mounted on a glass fiber. Diffraction data were collected on a Bruker SMART CCD 1K diffractometer. The frame data were acquired with the SMART /16a/ software at 298 K using Mo Kα radiation ($\lambda$ = 0.71073 Å). Final values of the cell parameters were obtained from least-squares refinement of the positions of all observed reflections. A total of 1271 thirty-second frames were collected in three sets with 0.3° ω-scan. The frames were then processed using the SAINT software /16b/ to give the *hkl* file corrected for Lorentz and polarization effects. A multi-scan absorption correction was applied. The structure was solved by direct method using the SHELX-90 /16c/ program and refined by least-squares method on $F^2$, SHELXTL-93 /16d/, incorporated in SHELXTL, Version 5.1 /16e/. The initial E-map yielded all non-hydrogen atom positions. Hydrogen atoms for carbons were geometrically positioned and left riding on their parent atoms during structure refinement. The hydrogen atoms for oxygens were located from the Fourier map, fixed at appropriate distances and let to ride on the parent oxygen atoms. The molecular structure of PBTC is shown in Figure 2 with 50 % probability ellipsoids. A view of a PBTC molecule connected to its neighbors through hydrogen bonds is shown in Figure 3. A packing diagram down the x-axis is shown in Figure 4.

The crystal of PBTC·$H_2O$ contains a molecule of $H_2O$ per asymmetric unit. Final positional parameters, along with their standard deviations as estimates from the inverse matrix are given in Table 2. Bond lengths in PBTC·$H_2O$ are given in Table 3 and angles are given in Table 4. Hydrogen bonds are given in Table 5. Crystallographic data for PBTC-$H_2O$ have been deposited with CCDC, with number 258094. Copies of the data can be obtained from deposit@ccdc.com.ac.uk or www.ccdc.com.ac.uk.

**Table 1**

Summary of Crystal Data, Intensity Collection and Structure Refinement Parameters for $PBTC\cdot H_2O$.

| | |
|---|---|
| Compound | $PBTC\cdot H_2O$ |
| Formula | $C_7H_{13}O_{10}P$ |
| Molecular Weight | 288.14 |
| a (Å) | 7.6711(8) |
| b (Å) | 8.6804(10) |
| c (Å) | 9.8856(11) |
| α (deg) | 65.518(2) |
| β (deg) | 71.683(2) |
| γ (deg) | 76.173(2) |
| V ($Å^3$) | 564.20(11) |
| Z | 2 |
| Crystal System | triclinic |
| Space Group | $P\bar{1}$ |
| Crystal Size (mm) | 0.26 x 0.22 x 0.14 |
| $d_{calcd}$ ($g/cm^3$) | 1.696 |
| Diffractometer | Bruker AXS SMART 1K CCD |
| Radiation | Mo Kα (λ=0.71073 Å) |
| Collection Temperature | 298(2) °C |
| Absorption coefficient □, $cm^{-1}$ | 0.292 |
| F(000) | 300 |
| $2\theta_{max}$ range (deg) | 4.66 to 55.94 |
| Total Reflections | 3505 |
| Independent Reflections | 2524 [*R*(int) = 0.0194] |
| Refined Reflections ($I_{net}>2\sigma I_{net}$) | 2273 [*R*(int) = 0.0188] |
| Refinement method | Full-matrix least squares on $F^2$ |
| Index ranges | $-9 \le h \le 10$, $-11 \le k \le 7$, $-12 \le l \le 12$ |
| Number of Parameters | 166 |
| R (%)[a] (R (%), all reflections) | 3.07 (3.40) |
| $R_w$ (%)[b] ($R_w$ (%), all reflections) | 8.48 (8.69) |
| Goodness of Fit[c] | 1.04 |
| Deepest Hole ($e/Å^3$) | -0.319 |
| Highest Peak ($e/Å^3$) | 0.327 |

[a] $R = \Sigma(|F_O-F_C|)/\Sigma|F_O|$ [b] $R_W = [\Sigma (w|F_O-F_C|)^2/\Sigma w(F_O)^2]^{1/2}$

[c] $GoF = [\Sigma w(F_O-F_C)^2/(\text{no. of reflections - no. of parameters})]^{1/2}$

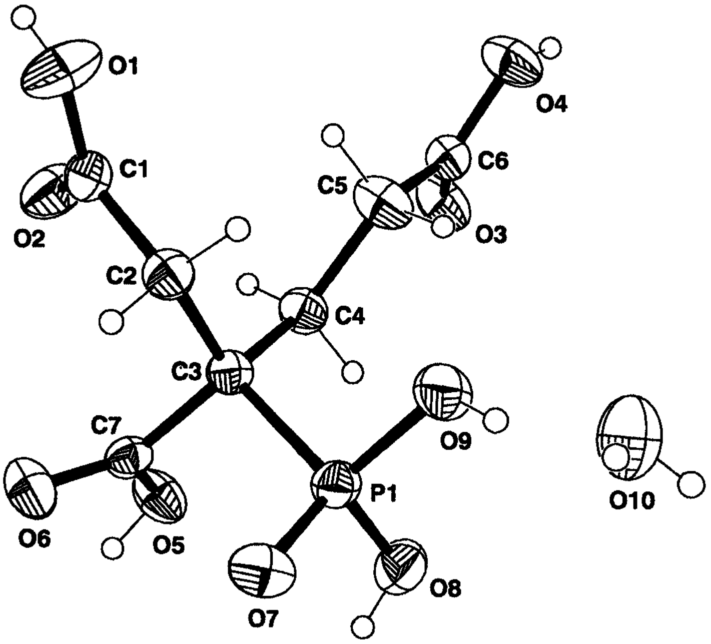

**Fig. 2:** ORTEP diagram of PBTC (50 % ellipsoids) with the water of crystallization.

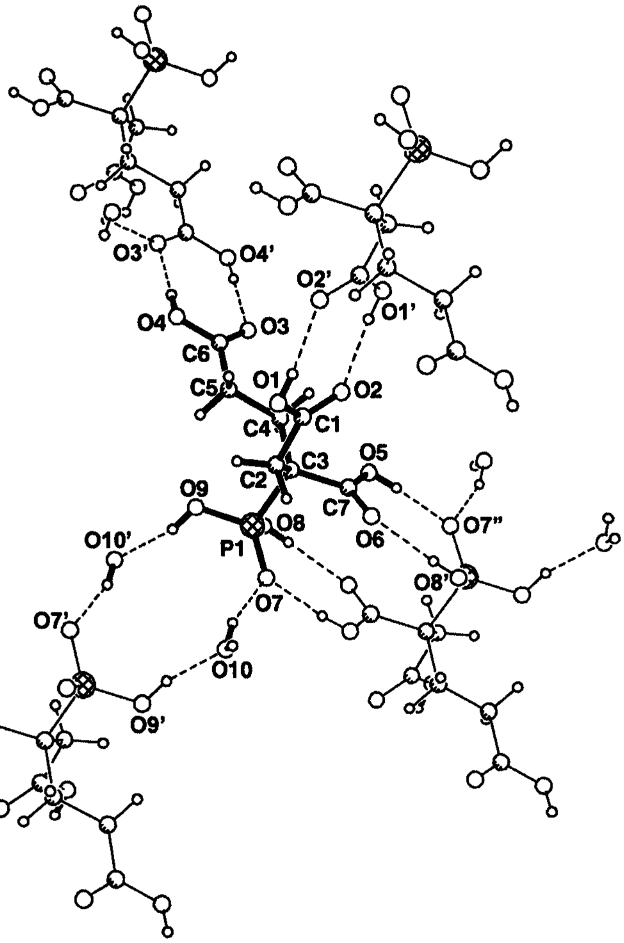

**Fig. 3:** A view of the PBTC·$H_2O$ structure showing all hydrogen bonds.

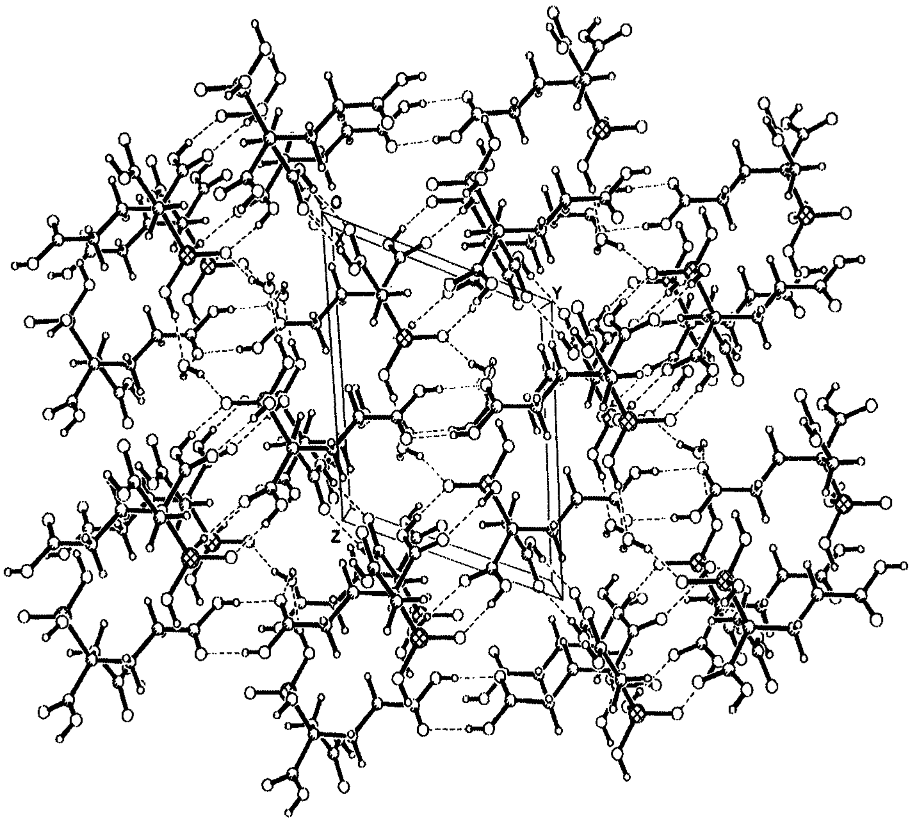

**Fig. 4:** A packing diagram of the PBTC·$H_2O$ structure down the x-axis.

### RESULTS AND DISCUSSION

### Crystal Structure and Lattice

The presence of protonated phosphonate and carboxylate groups as well as the water molecule creates an intricate network of hydrogen bonding. The complexity of the structure can be seen in Figures 3 and 4. Hydrogen bonding distances are reported herein as D···A (donor···acceptor) and H-bonds are given notations such as a, b, c, etc.

The -$PO_3H_2$ group acts as both donor and acceptor and forms two sets, a total of four, H-bonds. The first set forms between the -P=O (from one molecule), the –P-O(H) (from a neighboring molecule) and two water molecules of crystallization. This H-bonding mode forms an 8-member ring (not counting the H atoms) and is locally centrosymmetric. The O(9)-H(9)···O(10") distance (bond a) is rather short, 2.4622(14) Å. The O(9)-H(9)···O(10") angle is slightly bent, 164.8°. The $O_w$(10")-$H_w$(1)···O(7) distance (bond b) is much longer, 2.6736(15) Å. The $O_w$(10")-$H_w$(1)···O(7) angle is 161.6°.

The second set consists of two hydrogen bonds. These are formed between the P=O portion of the -$PO_3H_2$ group and the –OH group of the carboxylate of a neighboring molecule, and between the second –P-O(H) group and the carbonyl –C=O portion of the same carboxylate. This generates a 6-member ring (not counting

**Table 2**

Atomic Parameters x,y,z (x$10^4$) for PBTC·$H_2O$.

Estimated Standard Deviations (esd's) refer to the last digit printed.

| Atom | x | y | z |
|---|---|---|---|
| P(1) | 1983(1) | 6787(1) | 6992(1) |
| O(1) | 6270(2) | 8839(2) | 8665(2) |
| O(2) | 3351(2) | 9030(2) | 10025(2) |
| O(3) | -336(2) | 13121(2) | 6404(2) |
| O(4) | 2312(2) | 13816(2) | 4663(2) |
| O(5) | -652(2) | 7318(2) | 10192(2) |
| O(6) | 1871(2) | 5480(2) | 10672(2) |
| O(7) | 2687(2) | 4932(2) | 7570(2) |
| O(8) | -84(2) | 7080(2) | 6941(2) |
| O(9) | 3046(2) | 7859(2) | 5406(2) |
| C(1) | 4533(2) | 8587(2) | 9060(2) |
| C(2) | 4190(2) | 7681(2) | 8177(2) |
| C(3) | 2128(2) | 7837(2) | 8233(2) |
| C(4) | 1143(2) | 9707(2) | 7721(2) |
| C(5) | 2287(2) | 10967(2) | 6324(2) |
| C(6) | 1269(2) | 12728(2) | 5825(2) |
| C(7) | 1110(2) | 6768(2) | 9847(2) |
| $O_w$(10) | 4275(2) | 7162(2) | 3079(2) |

**Table 3**

Selected bond distances (Å) in PBTC·$H_2O$.

| Bond | Distance | Bond | Distance |
|---|---|---|---|
| P(1)-O(7) | 1.4928(10) | O(5)-C(7) | 1.3065(16) |
| P(1)-O(9) | 1.5294(10) | O(6)-C(7) | 1.2195(16) |
| P(1)-O(8) | 1.5578(10) | C(1)-C(2) | 1.5039(18) |
| P(1)-C(3) | 1.8465(12) | C(2)-C(3) | 1.5405(16) |
| O(1)-C(1) | 1.3101(17) | C(3)-C(7) | 1.5338(16) |
| O(2)-C(1) | 1.2146(18) | C(3)-C(4) | 1.5602(16) |
| O(3)-C(6) | 1.2151(17) | C(4)-C(5) | 1.5226(18) |
| O(4)-C(6) | 1.3180(16) | C(5)-C(6) | 1.4957(18) |

**Table 4**

Selected angles (deg) in PBTC·$H_2O$.

| Angle | Degrees | Angle | Degrees |
|---|---|---|---|
| O(7)-P(1)-O(9) | 115.97(6) | C(2)-C(3)-C(4) | 114.50(10) |
| O(7)-P(1)-O(8) | 111.44(6) | C(7)-C(3)-P(1) | 104.62(8) |
| O(9)-P(1)-O(8) | 106.99(6) | C(2)-C(3)-P(1) | 107.24(8) |
| O(7)-P(1)-C(3) | 111.46(6) | C(4)-C(3)-P(1) | 109.84(8) |
| O(9)-P(1)-C(3) | 103.17(6) | C(5)-C(4)-C(3) | 114.48(10) |
| O(8)-P(1)-C(3) | 107.15(6) | C(6)-C(5)-C(4) | 112.95(11) |
| O(2)-C(1)-O(1) | 123.82(13) | O(3)-C(6)-O(4) | 123.48(12) |
| O(2)-C(1)-C(2) | 124.37(12) | O(3)-C(6)-C(5) | 124.53(12) |
| O(1)-C(1)-C(2) | 111.81(12) | O(4)-C(6)-C(5) | 111.98(12) |
| C(1)-C(2)-C(3) | 113.18(10) | O(6)-C(7)-O(5) | 124.23(12) |
| C(7)-C(3)-C(2) | 109.40(10) | O(6)-C(7)-C(3) | 122.40(11) |
| C(7)-C(3)-C(4) | 110.72(10) | O(5)-C(7)-C(3) | 113.23(10) |

**Table 5**

Hydrogen Bonds in PBTC·$H_2O$ with H···A < r(A) + 2.000 Å and ∠ D-H-A > 110 deg.

| D-H | H···A | *D···A* | ∠ D-H-A (deg) | angle | symmetry operation |
|---|---|---|---|---|---|
| 0.82 | 1.95 | 2.7674(15) | 171.2 | O(1)-H(1)···O(2') | [-x+1, -y+2, -z+2 ] |
| 0.82 | 1.87 | 2.6903(14) | 177.0 | O(4)-H(4)···O(3') | [-x, -y+3, -z+1 ] |
| 0.82 | 1.81 | 2.6171(13) | 165.7 | O(5)-H(5)···O(7") | [-x, -y+1, -z+2 ] |
| 0.79 | 1.94 | 2.7319(14) | 171.1 | O(8)-H(8)···O(6') | [-x, -y+1, -z+2 ] |
| 0.82 | 1.67 | 2.4622(14) | 164.8 | O(9)-H(9)···O(10') | [x, y, z] |
| 0.82 | 1.89 | 2.6736(15) | 161.6 | $O_w$(10)-$H_w$(2)···O(7) | [-x+1, -y+1, -z+1 ] |
| 0.82 | 2.31 | 2.9552(17) | 135.6 | $O_w$(10)-$H_w$(2)···O(3) | [-x, -y+2, -z+1 ] |

the H atoms). The O(7)···H(5')-O(5') distance (bond c) is 2.6171(13) Å and the O(8)–H(8)···O(6)' bond distance is 2.7319(14) Å. The O(7)···H(5')-O(5') angle is 165.7° and the O(8)–H(8)···O(6)' angle is 171.1°.

The O(7) atom of the phosphonate –P=O group participates in two H-bonds and acts as a bridge between a water proton and a carboxylate proton. These bonds are dissimilar in length (2.6736(15) Å in the former and 2.7319(14) Å in the latter). It appears that the formation of the 4-member ring facilitates a shorter H-bond compared to that from an 8-member ring.

The –COOH group at the 2' position participates in hydrogen bonding interactions with the -$PO_3H_2$ group as described above.

The –COOH group at the 4' position forms a commonly seen "dicarboxylate dimer" with a neighboring carboxylate also at the 4' position. This "dimer" sits on a "local" inversion center. The bond distance is 2.6903(14) Å for O(4)-H(4)···O(3'). The O atom of the carboxylate C=O group also forms a very long H-bond with on of the water protons, at a distance of 2.9552(17) Å.

The –COOH group at the 1' position forms the aforementioned "dicarboxylate dimer" with a neighboring carboxylate (also at the 1' position) forming a 6-member ring. The H-bonding distance is 2.7674(15) Å.

Bond lengths are presented in Table 3. All three carboxylate and the phosphonate groups in PBTC are protonated. The P=O double bond length is 1.4928(10) Å, whereas the P-O single bonds are 1.5294(10) Å and 1.5578(10) Å. The P-C bond length is 1.8465(12) Å and it falls in the normal range (1.8-1.9 Å) for such bonds /8f,14,15/.

### Vibrational spectroscopy

A very broad and intense band appears around 1700 $cm^{-1}$ in the IR spectrum of PBTC (in KBr pellets). This is characteristic of uncoordinated, protonated carboxylate and is assigned to the C=O stretch of -COOH /17/. The width and asymmetry of this band indicates that it is mixed with the band arising from water of crystallization, and is consistent with the presence of extended hydrogen bonding.

Multiple bands appear in the region 900-1150 $cm^{-1}$ characteristic of $-PO_3$ stretching frequencies /18/. Multiple C-C vibrations appear around 1400 $cm^{-1}$. O-H stretching vibrations appear at 3400-3550 $cm^{-1}$ associated with –OH from phosphonate/carboxylate groups and water of crystallization. Assignments for the P-OH stretching vibration (around 880 $cm^{-1}$) and the bending deformation mode of $H_2O$ (around 660 $cm^{-1}$) were not possible due to the unusually high noise in that region of the spectrum. Our attempts to obtain better quality spectra were unsuccessful.

### Structural features of phosphonates: Literature examples

There exists a plethora of crystal and molecular structures of *mono*-phosph<u>i</u>nates /19/ and *mono*-phosph<u>o</u>nates /20/ in the crystallographic literature. In addition, an extensive number of structures of *bis*-phosphonates /21/ exist. Furthermore, *ab initio* studies on relevant organophosphorous compounds and their Ca complexes have been carried out /22/. Substantial interest has been focused on 1,1-*bis*-phosphonates (or *gem-bis*-phosphonates) because there are data available that point to their better performance as scale inhibitors than *mono*-phosphonates /23/. On the other hand, two neighboring (*geminal*) phosphonate groups are not a necessary requirement for good inhibition. AMP and PBTC are examples of excellent $CaCO_3$ inhibitors although they do not possess *gem-bis*-phosphonate groups /24/. A brief review of representative literature cases relevant to this study is presented below.

HEDP is one of the most widely used phosphonate in the field of water treatment. Its crystal structure as a monohyrdate consists of columns of symmetry-related HEDP molecules linked together by a complex hydrogen-bonding network of hydroxyl and phosphonic acid groups /21k/. All hydrogen-bonding is intermolecular. All four of phosphonic acid hydrogens are involved in hydrogen bonds. P-O(H) bond lengths are in the range 1.537-1.559 Å. P=O bond lengths are ~1.506 Å.

The crystal structure of the closely related methylenediphosphonic acid has been reported /21c/. Every phosphonyl hydrogen is asymmetrically bonded to two oxygen atoms to form an extensive three-dimensional network of hydrogen bonds involving all molecules of the unit cell. Each P=O oxygen is an acceptor of two intermolecular hydrogen bonds, while each P-O(H) oxygen is bonded to a single hydrogen.

A molecule of relevance is ethane-1,2-diphosphonic acid, which has two methylene groups (instead of one) separating the phosphonate groups /21b,c/. Molecules of ethane-1,2-diphosphonic acid pack within the unit cell in such a manner that the C-C bond is located at an inversion center. There is no intramolecular hydrogen bonding. Each hydrogen atom is asymmetrically bound to two oxygens, with P=O oxygens bonding to two hydrogens, as in the case with methylenediphosphonic acid.

An addition to the above pair of *bis*-phosphonates is the structure of propane-1,3-diphosphonic acid /21d/. It consists of two non-related and unique molecules that adopt different configurations. The atoms in each molecule are oriented to form P-C-C-C-P chains that are linked *via* hydrogen bonding (closest contacts 2.603(3) Å and 2.580(10) Å).

The molecular structure of a biologically active *bis*-phosphonate, 1-hydroxy-2,2,2-trifluoroethylidene-*bis*-phosphonic acid, was reported as its *tris*(trimethylsilyl) ester derivative /21a/. The perfluoroalkyl groups were introduced in order to increase its lipophilicity. Molecules of the perfluorophosphonate were found to arrange pair-wise in the unit cell *via* two P=O···HO-P and two P=O···HO-C hydrogen bonds.

The structures of two halo-*bis*-phosphonates were reported. These are *tetrakis*(1-methylethyl) (dichloromethylene)bisphosphonate, and tetramethyl (dibromomethylene) bisphosphonate /21j/ P-O and P-C bond lengths fall within the normal range.

The molecular and crystal structure of di-*t*-butylphosphinic acid was determined by single-crystal X-ray and neutron diffraction /19/ It reveals a dimeric arrangement in the solid state held by strong hydrogen bonds (2.506(18) Å). The 8-member ring that forms as a result of this intermolecular hydrogen bonding has $\bar{1}$ site symmetry and shows a small chair-conformation distortion from planarity. The P-O bond lengths are 1.521(8) Å and 1.520(6) Å, an indication that the O atoms are essentially equivalent. There also appear to be weak C-H···O interactions that fix the methyl group orientations resulting into an overall eclipsed conformation.

In the structure of tetrasodium carbonyldiphosphonate the presence of the C=O group forces the C-$(PO_3)_2$ group to be coplanar. The P-C-P and P-C-O angles are 120.7 and 119.6, respectively, statistically the same as the 120° ideal value. The P-C bond length is 1.874(1) Å /21l/.

### CONCLUSIONS

The crystal and molecular structure of 2-phosphonobutane-1,2,4-tricarboxylic acid monohydrate is reported. The water of crystallization and phosphonate and carboxylate groups participate in extensive intermolecular hydrogen bonding. Similar intermolecular hydrogen interactions have been observed in the structure of anilinium butanebisphosphonate /25/. The -$PO_3H_2$ group acts as both donor and acceptor and forms two sets, a total of four, H-bonds. The first set forms between the -P=O and –P-O(H) and the water of crystallization. The second set consists of two of hydrogen bonds between the P=O portion of the phosphate group and the –OH group of the carboxylate of a neighboring molecule, and between the second –P-O(H) group and the carbonyl –C=O portion of the same carboxylate.

In spite of intensive research the quest for the "ideal" scale inhibitor is an ongoing endeavor /26/. Such an inhibitor must possess the following highly desirable properties: (a) excellent scale inhibition performance, (b) high Ca tolerance (resistance to Ca-inhibitor salt precipitation), (c) stability towards oxidizing biocides, (d) thermal stability (for high temperature applications), and (d) low production cost.

Certain phosphonates used extensively in the water treatment industry have limitations. They are known to be susceptible to oxidation by chlorine or bromine-based biocides (necessary to control microbiological growth in industrial waters) /27/. HEDP, AMP and certain aminomethylene phosphonates are examples of this class. Orthophosphate ($PO_4^{3-}$), one of the final products of this oxidative degradation, can cause additional problems because of the potential to form calcium phosphate scale (in addition to the existing possibility of other scales). These phosphonates can also form precipitates with calcium that form deposits /28/. Therefore, phosphonates that are resistant to decomposition by oxidative biocides and exhibit high calcium tolerance are highly desirable. PBTC is virtually immune to biocides such as chlorine, bromine, hypochlorite and others, and does not decompose to any appreciable extent, at least at "normal" biocide dosage (see following paper). It also shows high calcium tolerance. These properties together with its excellent inhibition performance make PBTC a molecule of great interest and a subject of study in our group. Future studies will involve computational approaches to interactions of PBTC with sparingly soluble salt crystal surfaces. Such studies have been performed for interactions of phosphocitrate (a potent biomineralization inhibitor /29/) with calcium pyrophosphate dihydrate /30/ and hydroxyapatite surfaces /31/.

The systematic synthesis of a plethora of phosphonates and their metal salts, their structural characterization and potential applications of these novel structures are currently underway in our laboratory /32/.

### ACKNOWLEDGMENTS

We wish to thank Mr. Dionysios Halas of Solutia Inc. for providing the sample of PBTC.

### REFERENCES

1. *Calcium Carbonate*: (a) Koutsoukos, P.G.; Kontoyannis, C.G. *J. Chem. Soc. Faraday Trans.* **1984**, *80*, 1181. (b) Xyla, G.A.; Giannimaras, E.K.; Koutsoukos, P.G. *Colloids Surf.* **1991**, *53*, 241. (c) Gal, J.-Y.; Bollinger, J.-C.; Tolosa, H.; Gache, N. *Talanta* **1996**, *43*, 1497. (d) Van Der Weijden et al. *J. Cryst. Growth* **1997**, *171*, 190. (e) Gomez-Morales, J. et al. *J. Cryst. Growth* **1996**, *166*, 1020. (f) Zafiropoulou, A.; Dalas, E. *J. Cryst. Growth* **2000**, *219*, 477. (g) Dalas, E. *J. Cryst. Growth* **2001**, *222*, 287.
2. *Calcium Phosphates*: (a) Z. Amjad "Calcium Phosphates in Biological and Industrial Systems" Kluwer Academic Publishers: Boston 1998. (b) Koutsoukos, P.; Amjad, Z.; Tomson, M.B.; Nancollas, G.H. *J.*

*Am. Chem. Soc.* **1980**, *102*, 1553. (c) Sørensen, J.S.; Lundager Madsen, H.E. *J. Cryst. Growth* **2000**, *216*, 399. (d) Golubev, S.V.; Pokrovsky, O.S.; Savenko, V.S. *J. Cryst. Growth* **1999**, *205*, 354. (e) Deluchat, V.; Bollinger, J.-C.; Serpaud, B.; Caullet, C. *Talanta* **1997**, *44*, 897. (f) F. H. Browning, H.S. Fogler, *SPE Production & Facilities* **1995**, *August*, 144. (g) Amjad, Z. *Tenside Surf. Det.* **1997**, *34*, 102. (h) US patent 5,346,010. (i) Zieba, A.; Sethuraman, G.; Perez, F.; Nancollas, G.H.; Cameron, D. *Langmuir* **1996**, *12*, 2853. (j) US patent 5,211,237.

3. (a) Nancollas, G.H.; Klima, W.F. *Materials Performance* **1982**, *April, p. 9.* (b) Dalas, E.; Koutsoukos, P.G. *Geothermics* **1989**, *18*, 83. (c) Kim, W.T.; Bai, C.; Cho, Y.I. *Int. J. of Heat Mass Transfer* **2002**, *45*, 597. (d) Kim, W.T.; Cho, Y.I. *Int. Comm. Heat Mass Transfer* **2001**, *28*, 671. (e) Morizot, A.; Neville, A; Hodgkiess, T. *J. Cryst. Growth* **1999**, *198/199*, 738. (f) Cho, Y.; Choi, B.-G. *Int. J. of Heat Mass Transfer* **1999**, *42*, 1491. (g) Watkinson, A.P.; Martinez, O. *Trans. ASME* **1975**, *97*, 504. (h) Tatterson, G. *Chemical Processing* **2002**, *65 (January)*, 12. (i) *Fouling of Heat Transfer Equipment*; Somerscales, E. F. C., Knudsen, J. G., Eds.; Hemisphere Publishing Corp.: New York, 1981. (j) Hasson, D.; Averiel, M.; Resnick, W.; Rozemann, T.; Winderich, S. *Ind. Eng. Chem. Fundam.* **1968**, *7*, 59.
4. (a) S. Zaheer Akhtar, *Power Engineering* **2000**, *October*, 63. (b) J. Katzel, *Plant Engineering* **1989**, *27 (April)*, 32. (c) Power Special Report, *Power* **1973**, *March*, S-1. (d) E.C. Elliot, *Power* **1985**, *December*, S-1. (e) R. Burger, *American Power Conference* **1995**, *Vol. 57*, p. 1363. (f) R. Burger, *American Power Conference* **1994**, *Vol. 56*, p. 1085.
5. (a) "The Chemistry of Silica" Iler, R.K. Wiley & Sons: New York, 1979. (b) Young, P.R.; Stuart, C.M.; Eastin, P.M. *Cooling Technology Institute 1993 Annual Meeting, Technical Paper # TP93-11.* (c) Dubin, L. US Patents 4,532,047 and 4,584, 104. (d) Meier, D.A.; Dubin, L. Paper No. 334, National Association of Corrosion Engineers, Houston, TX, 1987. (e) Gill, J.S. *Materials Performance* **1998**, *November, p. 41.* (f) Dubin, L.; Dammeier, R.L.; Hart, R.A. *Materials Performance* **1985**, *October, p. 27.* (g) Young, P.R. Paper No. 466, National Association of Corrosion Engineers, Houston, TX, 1993.
6. (a) *Mineral Scale Formation and Inhibition,* Amjad, Z. Ed.; Plenum Press: New York, 1995 and references therein. (b) P. Ashdown, D. Ashworth, W. Guthrie, *Performance Chemicals Europe* **1999**, *November/December*, 28. (c) Matty, J.M.; Tomson, M.B. *Appl. Geochem.* **1988**, *3*, 549. (d) Tomson, M.B. *J. Cryst. Growth* **1983**, *62*, 106.
7. (a) Amjad, Z.; Pugh, J.; Zibrida, J.; Zuhl, B. *Materials Performance* **1997**, *January*, p. 32. (b) Rieger, J.; Hadicke, E.; Rau, I.U.; Boeckh, D. *Tenside Surf. Det.* **1997**, *6*, 430. (c) Amjad, Z. *Tenside Surf. Det.* **1997**, *34*, 102. (d) J.E. Hoots, G.A. Crucil, Corrosion/86, Paper No. 13, National Association of Corrosion Engineers, Houston, TX, 1986. (e) E.B. Smyk, J.E. Hoots, K.P. Fivizzani, K.E. Fulks, Corrosion/88, Paper No. 14, National Association of Corrosion Engineers, Houston, TX, 1988. (f) C.C. Pierce, J.E. Hoots in Chemical Aspects of Regulation of Mineralization, C.S. Sikes and A.P. Wheeler Eds., University of Alabama Publication Services, Alabama 1988, p. 53. (g) C.C. Pierce, D.A. Grattan, Corrosion/88, Paper No. 205, National Association of Corrosion Engineers, Houston, TX, 1988.
8. (a) Dequest: Phosphonates by Solutia (2054 Phosphonates for scale and corrosion control, chelation, dispersion), Publication # 7450006A. (b) Dequest: Phosphonates by Solutia (2060-S, 2066 & 2066-A Phosphonates: metal ion control agents), Publication # 7459369. (c) Dequest: Phosphonates by Solutia (Introductory Guide), Publication # 7459151B. (d) Dequest: Phosphonates by Solutia (2000 & 2006

Phosphonates for scale and corrosion control, chelation, dispersion), Publication # 7459023B. (e) Silvestre, J.-P.; Dao, N. Q.; Leroux, Y. *Heteroatom Chemistry*, **2001**, *12*, 73, supplement to the Special Issue on the chemistry and biochemistry of bisphosphonates and aminophosphonates, **2000**, *Vol. 11*, Issue 7. (f) Silvestre, J.-P.; N. Q. Dao, N. Q.; Salvini, P. *Phosphorus Sulfur Silicon* **2002**, *177*, 771.

9. (a) Bosbach, D.; Hochella, M.F. *Chemical Geology* **1996**, *132*, 277. (b) Amjad, Z. *Langmuir* **1991**, *7*, 600. (c) Fulks, K.E.; Yeoman, A.M. Corrosion/83, Paper No. 279, National Association of Corrosion Engineers, Houston, TX, 1983. (d) Hale, E.R.; Hoots, J.E.; Nicolich, S.N. *Power Engineering* **1999**, September, 21. (e) Amjad, Z.; *Can. J. Chem.* **1988**, *66*, 2180.
10. (a) Sweeney, F.M.; Cooper, S.D. *Society of Petroleum Engineers International Symposium on Oilfield Chemistry, New Orleans, LA March 2-5, 1993, paper SPE 25159.* (b) Oddo, J.E.; Tomson, M.B. Corrosion/92, Paper No. 34, National Association of Corrosion Engineers, Houston, TX, 1992. (c) Xiao, J.; Kan, A.T.; Tomson, M.B. *American Chemical Society-Division of Fuel Chemistry, Symposium Preprints* **1998**, *43*, 246. (d) Oddo, J.E.; Tomson, M.B. *SPE Production & Facilities* **1994**, *February*, 47. (e) Browning, F.H.; Fogler, H.S. *AIChE Journal* **1996**, *42*, 2883.
11. (a) Darling, D.; Rakshpal, R. *Materials Performance* **1998**, *December*, 42. (b) Strauss, S.D. *Power* **1992**, *September*, 17. (c) Farooqi, I.H.; Nasir, M.A.; Quraishi, M. *Corr. Prev. & Control* **1997**, *October*, 129. (d) Andijani, I.; Turgoose, S. *Desalination* **1999**, *123*, 223.
12. (a) Demadis, K.D.; Yang, B.; Young, P.R.; Kouznetsov, D.L.; Kelley, D.G. in *Advances in Crystal Growth Inhibition Technologies*; Amjad, Z., Editor; Plenum Press, New York: **2000**, Chapter 16, p. 215. (b) Sallis, J. D. In *Calcium Phosphates in Biological and Industrial Systems*; Amjad, Z., Ed.; Kluwer Academic Publishers: New York, 1998; Chapter 8, p 173. (c) Amjad Z. in Amjad Z, Ed. *Mineral Scale Formation And Inhibition*, New York: Plenum Press, 1995. p. 207. (d) Dubin, L. Corrosion/80, Paper No. 222, National Association of Corrosion Engineers, Houston, TX, 1980. (e) Ashdown, P.; Ashworth, D.; Guthrie, W. *Performance Chemicals Europe* **1999**, *November/December*, 28.
13. (a) Oddo, J.E.; Tomson, M.B. *SPE Production & Facilities* **1994**, *February*, 47. (b) Oddo, J.E.; Tomson, M.B. Paper No. 34, National Association of Corrosion Engineers, Houston, TX, 1992. (c) Sarig, S.; Ginio, O. *J. Phys. Chem.* **1976**, *80*, 256. (d) Oddo, J.E.; Tomson, M.B. *SPE Production & Facilities* **1994**, *February*, 47. (e) Cowan, J.C.; Weintritt, D.J. *Water-Formed Scale Deposits*, Gulf Publishing Co. Houston, TX, 1976, p. 93.
14. (a) Cao, G.; Hong, H.-G.; Mallouk, T. E. *Acc. Chem. Res.* **1992**, *25*, 420 (b) Malllouk, T. E.; Kim, H. N.; Olliver, P. J.; Keller, S. W. In *Comprehensive Supramolecular Chemistry*; Alberti, G., Bein, T., Eds.; Pergamon: New York, 1996; Vol. 7, pp. 189. (c) Alberti, G. In *Comprehensive Supramolecular Chemistry*; Alberti, G., Bein, T., Eds.; Pergamon: New York,1996; Vol. 7, pp 151. (d) Clearfield, A. *Prog. Inorg. Chem.***1998**, *47*, 371. (d) Clearfield, A.; Krishnamohan Sharma, C. V.; Zhang, B. *Chem. Mater.* **2001**, *13*, 3099.
15. (a) Dines, M. B.; Cooksey, R. E.; Griffith, P. C.; Lane, R. H. *Inorg. Chem.* **1983**, *22*, 1003. (b) Yang, H. C.; Aoki, K.; Hong, H.-G.; Sackett, D. D.; Arendt, M. F.; Yau, S.-L.; Bell, C. M.; Mallouk, T. E. *J. Am. Chem. Soc.* **1993**, *115*, 11855. (c) Penicaud, V.; Massiot, D.; Gelbard, G.; Odobel, F.; Bujoli, B. *J. Mol. Struct.* **1998**, *470*, 31. (d) Serre, C.; Ferey, G. *Inorg. Chem.* **1999**, *38*, 5370. (e) Serpaggi, S.; Ferey, G. *J. Mater. Chem.* **1998**, 8, 2749. (f) Distler, A.; Lohse, D. L.; Sevov, S. C. *J. Chem. Soc., Dalton Trans.*

**1999**, 1805. (g) Poojary, D. M.; Zhang, B.; Clearfield, A. *J. Am. Chem. Soc.* **1997**, *119*, 12550. (h) Poojary, D. M.; Zhang, B.; Belling-Hausen, P.; Clearfield, A. *Inorg. Chem.* **1996**, *35*, 4942. (i) Alberti, G.; Vivani, R.; Murcia Mascaros, S. *J. Mol. Struct.* **1998**, *470*, 81. (j) Alberti, G.; Marcia-Mascaros, S.; Vivani, R. *J. Am. Chem. Soc.* **1998**, *120*, 9291.

16. (a) *SMART-NT Software Reference Manual,* version 5.059; Bruker AXS, Inc.: Madison, WI, 1998. (b) *SAINT+ Software Reference Manual,* version 6.02; Bruker AXS, Inc.: Madison, WI, 1999. (c) Sheldrick, G.M. *SHELXS-90, Program for the Solution of Crystal Structure*; University of Göttingen: Germany, 1986. (d) Sheldrick, G.M. *SHELXL-97, Program for the Refinement of Crystal Structure*; University of Göttingen: Germany, 1997. (e) *SHELXTL-NT Software Reference Manual,* version 5.1; Bruker AXS, Inc.: Madison, WI, 1998.
17. Demadis, K.D.; Coucouvanis, D. *Inorg. Chem.* **1995**, 34, 436.
18. Demadis, K.D.; Katarachia, S. *Phosphorus Sulfur Silicon* **2004**, 179, 627.
19. (a) Reis, A.H. Jr.; Peterson, S.W.; Dryan, M.E.; Gebert, E.; Mason, G.W.; Peppard, D.F. *Inorg. Chem.* **1976**, *15*, 2748. (b) Dryan, M.E.; Reis, A.H. Jr.; Gebert, E.; Peterson, S.W.; Mason, G.W.; Peppard, D.F. *J. Am. Chem. Soc.* **1976**, *98*, 4801.
20. (a) Cao, G.; Lynch, V.M.; Swinnea, J.S.; Mallouk, T.E. *Inorg. Chem.* **1990**, *29*, 2112. (b) Smith, P.H.; Raymond, K.N. *Inorg. Chem.* **1988**, *27*, 1056. (c) Gibson, D.; Karaman, R. *J. Chem. Soc. Dalton Trans.* **1989**, 1911. (d) Langley, K.J.; Squattrito, P.J.; Adani, F.; Montoneri, E. *Inorg. Chim. Acta* **1996**, *253*, 77. (e) Cao, G.; Lee, H.; Lynch, V.M.; Mallouk, T.E. *Solid State Ionics* **1988**, *26*, 63.
21. (a) Schloth, R.-M.; Lork, E.; Seifert, F.U.; Röschenthaler, G.-V.; Cohen, H.; Golomb, G.; Breuer, E. *Naturwissenschaften* **1996**, *83*, 571. (b) DeLaMatter, D.; McCullough, J.J.; Calvo, C. *J. Phys. Chem.* **1973**, *77*, 1146. (c) Peterson, S.W.; Gebert, E.; Reis, A.H. Jr.; Dryan, M.E.; Mason, G.W.; Peppard, D.F. *J. Phys. Chem.* **1977**, *81*, 466. (d) Gebert, E.; Reis, A.H. Jr.; Dryan, M.E.; Peterson, S.W.; Mason, G.W.; Peppard, D.F. *J. Phys. Chem.* **1977**, *81*, 471. (e) Sergienko, V.S.; Tolkacheva, E.O.; Ilyukhin, A.B. *Zh. Neorg. Khim.* **1993**, *38*, 1129. (f) Shkol'nikova, L.M.; Afonin, E.G.; Kalugina, E.V.; Sotman, S.S. *Kristallografiya* **1991**, *36*, 77. (g) Rochdaoui, R.; Silvestre, J.P.; Nguyen, Q.D.; Lee, M.R.; Neuman, A. *Acta Crystallog. Sect. C Cryst. Struct. Comm.* **1990**, *C46*, 2083. (h) Silvestre, J.P.; El Messbahi, N.; Rochdaoui, R.; Nguyen, Q.D.; Lee, M.R.; Neuman, A. *Acta Crystallog. Sect. C Cryst. Struct. Comm.* **1990**, *C46*, 986. (i) Afonin, E.G.; Pechurova, N.I.; Gladkikh, O.P.; Masyuk, A.A.; Shkol'nikova, L.M. *Zh. Obshch. Khim.* **1988**, *58*, 2646. (j) Barnett, B.L.; Strickland, L.C. *Acta Crystallog. Sect. B* **1979**, *B35*, 1212. (k) Uchtman, V.A.; Gloss, *J. Phys. Chem.* **1972**, *76*, 1298. (l) V.A. Uchtman, R.J. Jandacek *Acta Cryst.* **1976**, *B32*, 488.
22. (a) Björkroth, J.-P.; Peräkylä, M.; Pakkanen, T.A.; Pohjala, E. *J. Comput.-Aided Mol. Des.* **1992**, *6*, 303. (b) Peräkylä, M.; Pakkanen, T.A.; Björkroth, J.-P.; Pohjala, E. *J. Chem. Soc. Perkin Trans. 2* **1992**, 1167. (c) Räsänen, J.P.; Peräkylä, M.; Pohjala, E. Pakkanen, T.A. *J. Chem. Soc. Perkin Trans. 2* **1994**, 1055. (d) Räsänen, J.P.; Pohjala, E. Pakkanen, T.A. *J. Chem. Soc. Perkin Trans. 2* **1994**, 2485. (e) Räsänen, J.P.; Pohjala, E.; Pakkanen, T.A. *J. Chem. Soc. Perkin Trans. 2* **1995**, 39. (f) Räsänen, J.P.; Pohjala, E.; Nikander, H.; Pakkanen, T.A. *J. Phys. Chem.* **1996**, *100*, 8230. (g) Räsänen, J.P.; Pohjala, E.; Nikander, H.; Pakkanen, T.A. *J. Phys. Chem. A* **1997**, *101*, 5196.
23. (a) U.S. Patent 5,259,974. (b) European Patent Application 0 654 248 A1.

24. Oddo, J.E.; Tomson, M.B. *SPE Production & Facilities* **1994**, *February*, 47.

25. Mahmoudkhani, A.H.; Langer, V. *Cryst. Growth Des.* **2002**, *2*, 21.

26. Vanderpool, D. *International Water Conference 1997, paper # 40*, p. 383.

27. (a) Vaska, M. *Industrial Water Treatment* **1993**, *March/April*, p. 39. (b) Johnson, D.A.; Fulks, K.E.; Meier, D.A. Corrosion/86, Paper No. 403, National Association of Corrosion Engineers, Houston, TX, 1986. (c) European Patent Application 0 569 220 A2. (d) Berg, D.; Vanderpool, D.; Rubin, D. *International Water Conference 1987, paper # 7*, p. 56.

28. (a) Browning, F.H.; Fogler, H.S. *AIChE Journal* **1996**, *42*, 2883. (b) Amjad, Z. *Tenside Surf. Det.* **1997**, *34*, 102. (c) US patent 4,756,881. (d) Browning, F.H.; Fogler, H.S. *SPE Production & Facilities* **1995**, *August*, 144.

29. (a) Demadis, K.D.; Sallis, J.D.; Raptis, R.G.; Baran, P. *J. Am. Chem. Soc.* **2001**, *123*, 10129. (b) Demadis, K.D. *Inorg. Chem. Comm.* **2003**, 6, 527. (c) Sun, Y.; Reuben, P.; Wegner, L.; Sallis, J.D.; Demadis, K.D.; Cheung, H.S. *Frontiers in Biosciences*, **2005**, 10, 803.

30. Wierzbicki, A.; Cheung, H.S. *J. Mol. Struct.: THEOCHEM* **1998**, *454*, 287.

31. Wierzbicki, A.; Cheung, H.S. *J. Mol. Struct.: THEOCHEM* **2000**, *529*, 73.

32. (a) Demadis, K.D. in *Compact Heat Exchangers and Enhancement Technology for the Process Industries*; Editor: Shah, R., Begell House, New York, **2003**, *p.* 483. (b) Demadis, K.D.; Kataracbia, S.D.; Koutmos, M., *Inorg. Chem. Comm.*, **2005**, 8, 254. (c) Demadis, K.D.; Mantzaridis, C.; Raptis, R.G.; Mezei, G., *Inorg. Chem.*, **2005**, 44, 4469.

